# *Theobroma cacao* Virome: Exploring Public RNA-Seq Data for Viral Discovery and Surveillance

**DOI:** 10.3390/v17050624

**Published:** 2025-04-26

**Authors:** Gabriel Victor Pina Rodrigues, João Pedro Nunes Santos, Lucas Yago Melo Ferreira, Lucas Barbosa de Amorim Conceição, Joel Augusto Moura Porto, Eric Roberto Guimarães Rocha Aguiar

**Affiliations:** 1 Department of Biological Science, Center of Biotechnology and Genetics, Universidade Estadual de Santa Cruz, Ilhéus 45662-900, BA, Brazil; gvprodrigues.ppggbm@uesc.br (G.V.P.R.); jpnsantos.bio@uesc.br (J.P.N.S.); lucasmelobiomed@gmail.com (L.Y.M.F.); lucasbarbosa1714@gmail.com (L.B.d.A.C.); joelaugustomp@gmail.com (J.A.M.P.); 2 Department of Engineering and Computing, Universidade Estadual de Santa Cruz, Ilhéus 45662-900, BA, Brazil

**Keywords:** cocoa, Badnavirus, metatranscriptome, metavirome, mycoviruses

## Abstract

Cocoa (*Theobroma cacao* L.) is a major agricultural commodity, essential for the global chocolate industry and the livelihoods of millions of farmers. However, viral diseases pose a significant threat to cocoa production, with Badnavirus species causing severe losses in Africa. Despite its economic importance, the overall virome of *T. cacao* remains poorly characterized, limiting our understanding of viral diversity and potential disease interactions. This study aims to assess the cocoa-associated virome by analyzing 109 publicly available RNA-seq libraries from nine BioProjects, covering diverse conditions and geographic regions. We implemented a comprehensive bioinformatics pipeline integrating multiple viral sequence enrichment steps, a hybrid assembly strategy using different assemblers, and sequence similarity searches against NCBI non-redundant databases. Our approach identified ten putative novel viruses associated with the cocoa microbiome and a novel Badnavirus species. These findings provide new insights into the viral landscape of *T. cacao*, characterizing the diversity of cacao-associated viruses and their potential ecological roles. Expanding the catalog of viruses associated with cocoa plants not only enhances our understanding of plant–virus–microbiome interactions but also contributes to the development of more effective disease surveillance and management strategies, ultimately supporting sustainable cocoa production.

## 1. Introduction

Cocoa (*Theobroma cacao* L.) is a tropical plant classified under the Malvaceae family and the Theobroma genus [1]. Theobroma comprises 22 species, but *Theobroma cacao* L. stands out as the most significant and widely cultivated, primarily for its highly valued seeds [2]. *T. cacao* is not just the backbone of the chocolate industry but also a vital agricultural commodity that sustains millions of livelihoods worldwide [1,3]. Each year, approximately 4.5 million tons of cocoa are produced, generating over USD 10 billion in income for farming families [4]. For many smallholder farmers, particularly in West Africa, cocoa is more than a cash crop; it is the foundation of their economic survival. Countries like Côte d’Ivoire and Ghana lead global production [5,6], making cocoa a critical driver of employment, foreign exchange earnings, and economic stability in the region [7,8].

Traditionally known for its role in chocolate production, cocoa is being explored for various other industrial applications. By-products like cocoa shells and pod husks are increasingly used in animal feed, cosmetics, and even biofuels, adding both economic and environmental value to the crop [9,10]. As global demand for cocoa-based products continues to grow, particularly for chocolate, its economic significance remains as strong as ever [11].

Nevertheless, the industry faces mounting challenges. Climate change, market fluctuations, and sustainability concerns threaten cocoa production and pricing, putting pressure on the millions who depend on it [8,12]. In this scenario, one of the most destructive pathogens affecting *T. cacao* is *Moniliophthora perniciosa*, a hemibiotrophic fungus responsible for witches’ broom disease, which significantly disrupts cocoa production [13,14,15,16]. Another major threat is *Moniliophthora roreri*, which specifically infects developing cocoa pods, leading to frosty pod rot and further compromising yields [17].

Apart from these fungal infections, virus-related diseases represent an additional major threat to *T. cacao* and global agriculture, with far-reaching consequences for crop yield, quality, and plant health [18,19]. These viruses hijack plant cellular processes to replicate and spread, often resulting in symptoms such as stunted growth, necrosis, leaf and pod deformation, and discoloration [20]. For cocoa specifically, viral infections can directly reduce bean production and quality, disrupting supply chains and threatening economic stability [21,22,23]. This is particularly concerning in Africa, where the *Cacao swollen shoot virus* (CSSV) has devastated vulnerable cocoa varieties, particularly in West Africa [24,25,26]. Member of the Badnavirus genus within the *Caulimoviridae* family, this virus was first identified in Ghana in 1936 and has since spread to other cocoa-producing countries in West Africa, including Côte d’Ivoire, Nigeria, Togo, and Benin [27,28,29]. It is primarily transmitted by mealybugs, which facilitate its spread among cocoa trees [30]. Infected trees exhibit severe symptoms, including shoot swelling, yellowing leaves, stunted growth, and, in many cases, premature tree death [31,32].

Several viral infections have been documented in cocoa production, predominantly involving members of the Badnavirus genus, such as *Cacao mild mosaic virus* (CaMMV), *Cacao yellow vein banding virus* (CYVBV) [33], *Cacao red vein virus* (CRVV) [34], and many different isolates of CSSV [28,35]. However, other viral families have also been reported infecting *T. cacao*, including the *Solemoviridae* family, represented by *Cacao leafroll virus* from the Polerovirus genus, and the *Tymoviridae* family, which includes *Cacao yellow mosaic virus* (CYMV) [36]. These other species may cause several losses to cocoa production; CaMMV alone has been responsible for lower annual yield ranging from approximately 7% to 33% in infected plants [37].

Cocoa viral infections are widespread and exhibit substantial genetic diversity across different regions, posing significant challenges for disease control [35,38]. In Ghana, CSSV management has traditionally relied on practices such as removing infected trees and replanting to eliminate viral reservoirs [39]. However, no effective treatments are currently available for viral infections in cocoa trees, underscoring the urgency of improved disease management strategies. Addressing these threats is essential not only for agricultural sustainability but also for preserving economic stability and international trade networks [40].

This study aims to analyze metatranscriptomic data from publicly available *Theobroma cacao* RNA-seq libraries across various regions worldwide, providing information regarding the circulation of known pathogenic viruses and the detection of new emergent species.

## 2. Materials and Methods

### 2.1. Acquisition of RNA-Seq Libraries

The RNA library accessions were retrieved from the Sequence Read Archive (SRA) database available on the NCBI platform. The search was conducted using the keywords “Theobroma” AND “cacao”. Only RNA-seq libraries of *T. cacao* sequenced using the ILLUMINA^®^ platform were selected, encompassing both *single-end* and *paired-end* layouts. Libraries corresponding to technical replicates were excluded from the selection process. The BioProjects associated with the selected libraries were PRJEB35419, PRJNA604260, PRJNA613342, PRJNA742476, PRJNA785999, PRJNA558793, PRJNA314774, PRJNA421343, and PRJNA326055 (Supplementary Table S1).

### 2.2. RNA-Seq Processing and Assembly

Publicly available RNA-seq libraries were preprocessed with Fastp (version 0.23.2) [41] to eliminate adapter sequences and low-quality reads. Host-derived reads were filtered out by aligning the processed reads to the respective reference genome, Criollo cocoa B97-61/B2 (GCF_000208745.1) [42], using the STAR aligner (version 2.7.11b) [43] with default parameters. Unmapped reads were subsequently used for de novo assembly, employing two distinct tools: SPAdes (version 4.0.0) [44] using default settings and the transcripts derived by SPAdes were further processed using CAP3 (VersionDate: 2 October 2015) [45].

### 2.3. RNA-Seq-Based Assessment of the Cocoa Microbiome

The unmapped reads were analyzed for taxonomic classification by comparing them with the reference database PlusPFP (version: 7 June 2022) on Kraken2 (Galaxy Version 2.1.3+galaxy1) [46]. Kraken-report (Galaxy Version 1.3.1) was further applied to generate a report file using the Minikraken database from which we extracted and refined our results.

### 2.4. Metaviromic Analysis

Viral contigs were identified through sequence similarity searches using Diamond (version 2.1.8) in blastX mode [47] against a local nr database (release 08/2024) and BLAST (version 2.12.0+) [48] alignment with a local nt database (release 08/2024). The identified assembled contigs were filtered based on a minimum threshold of 500 nucleotides. The largest open reading frames (ORFs) within each contig were predicted using ORFiPy (version 0.0.4) [49], and the resulting sequences were further analyzed with HMMER (version 3.4) [50], using the *hmmsearch* application, against the Reference Viral Database (RVDB) HMM profile [51], version 29.0, and conserved domains were predicted using Protein Family Database (Pfam) [52] release 37.2.

### 2.5. Novel Virus Definition

Virus classification was based on the latest ICTV criteria, along with phylogenetic placement, genome organization, and pairwise protein identity with defined viruses. Specifically, viral species demarcation followed ICTV criteria, whereas viruses belonging to families or genera without established demarcation criteria were classified based on a nucleotide identity threshold of 90%. Similarly, genus and family classification adhered to the latest ICTV guidelines.

### 2.6. Integrative Genome Assembly

In order to improve possible viral genomes, an integrative assembly strategy was also performed by running the following different tools: rnaviralSPAdes (version 4.0.0) [44], Megahit (version 1.2.9) [53], and Trinity (version 2.15.2) [54], all assemblers with default parameters. The results were consolidated using CD-HIT (version 4.8.1) [55] to join sequences with more than 90% similarity. The assembled sequences were manually investigated and used along with raw read alignment to correct misassembled regions. Manually curated viral sequences identified in our work can be found in the Supplementary Table S2.

### 2.7. Molecular Phylogeny of Virus-Derived Sequences

Sequences exhibiting similarity to genes encoding RNA polymerase and polyproteins were used to construct phylogenetic trees. Additionally, protein sequences representing specific phyla, including Kitrinoviricota, Pisuviricota, Lernaviricota, and Duplornaviricota, as well as the *Caulimoviridae* viral family, were selected based on their proximity and references from the International Committee on Taxonomy of Viruses (ICTV). For each set of sequences, a global alignment was performed using MAFFT v7.505 [56] to ensure accurate alignment. The alignment was visualized and trimmed with AliView [57] version 1.29. Phylogenetic trees were then inferred using IQ-TREE [58] version 2.1.4 on a local server, employing the maximum likelihood method with 1,000 bootstrap replicates to evaluate the robustness of the tree topology. The trees were visualized in the online tool Interactive Tree of Life (ITOL v6) [59]. All novel viral sequences are available in Supplementary Table S2.

### 2.8. RNA Abundance and the Widespread Presence of Viral Segments

The transcriptional activity of virus-derived sequences was evaluated using Salmon (version 1.10.3) [60]. To compare viral abundance, the host NADH(1-4) and COX2 genes were selected as endogenous and standard reference genes, respectively. A summary of the transcriptional activity of the viral sequences included in this analysis is provided in Supplementary Table S3.

## 3. Results

### 3.1. Theobroma cacao Microbiome Assessment

**Figure 1:**
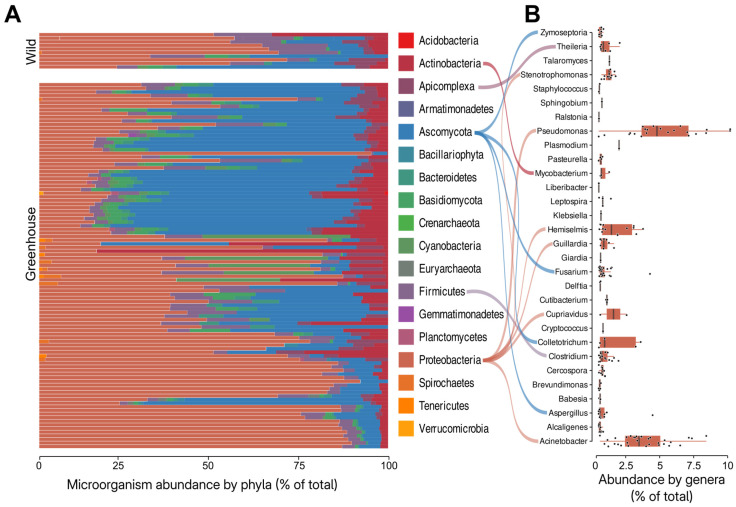
(**A**). Microbiome composition across the 109 *Theobroma cacao*-derived RNA-seq libraries, showing the relative abundance of the most frequent phyla. Phyla are represented only if they account for ≥0.05% of the total reads in each library. The *X*-axis indicates the relative number of reads classified within each phylum. (**B**). Reads abundance (%) in total metatranscriptome of the most frequent genera across all samples, considering only genera that account for ≥0.5% of the total reads in each library.

A total of 5,192,778,743 reads were obtained from all 109 *Theobroma cacao* RNA-seq libraries. Removal of host-derived reads led to 319,168,768 reads that were further classified using the microbiome composition-focused tool. The microbial diversity across all samples comprised 4577 species from 1428 genera, representing 452 distinct families. Elements from the phyla Proteobacteria and Ascomycota were the most abundantly represented and frequently detected in the cocoa RNA-seq libraries, followed by Actinobacteria and Firmicutes (Figure 1).

The geographic origin of each RNA-seq library spans a diverse range of regions worldwide. Among the libraries, 18 lack associated geographical metadata (from BioProjects PRJEB35419 and PRJNA314774), while the remaining 91 are derived from 10 countries: China, India, Colombia, the United States, Ecuador, Trinidad and Tobago, French Guiana, Brazil, Samoa, and Peru (Supplementary Table S1).

A subset of eight libraries, identified as wild samples based on NCBI SRA metadata (SRR3217278, SRR3217279, SRR3217280, SRR3217292, SRR3217281, SRR3217282, SRR3217283, and SRR3217284), originates from Ecuador, Colombia, and French Guiana. Analysis of species relative abundance revealed no distinct microbiome distribution patterns between these wild samples and those derived from greenhouse conditions (Supplementary Figure S1).

To further investigate geographic variations in species relative abundance, we compared samples based on their continent of origin (the Americas, Asia, and Oceania). A *t*-distributed stochastic neighbor embedding (*t*-SNE) analysis was performed to visualize the high-dimensional data structure. Samples from North and South America showed no clear separation, whereas libraries from Asia formed a distinct cluster. Additionally, libraries with unknown geographic metadata are grouped into a separate cluster, primarily composed of samples from the PRJEB35419 BioProject (Supplementary Figure S1).

### 3.2. Theobroma cacao-Associated Virome

We identified a considerable number of viral sequences in the metatranscriptomic analysis, prompting a deeper investigation into the presence of viruses in *Theobroma cacao* samples. After manual curation of de novo assembled transcripts, 34 non-redundant putative viral sequences longer than 500 nt were selected for further characterization. Sequence similarity searches against the NCBI non-redundant databases revealed 14 transcripts closely related to previously described viruses, while the remaining 20 sequences likely represent new viral species.

**Figure 2:**
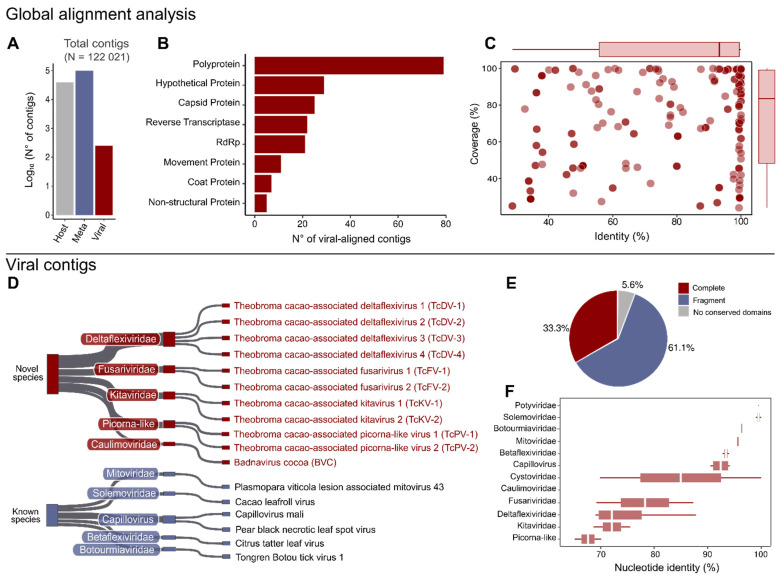
Metavirome overview. (**A**) Total contigs (N) from the de novo assembly of 109 cocoa RNA-seq libraries, classified as Meta, Host, or Viral based on protein alignment. Host material refers to residual reads that did not map to the reference genome. (**B**) Alignment targets of viral contigs based on protein alignment. (**C**) Identity and coverage of contigs aligned to viral protein sequences. (**D**) Classification of known and novel viruses by family and genetic material, as characterized in the contigs. (**E**) Distribution of complete, fragmented, and absent viral conserved domains in the characterized sequences. (**F)** Nucleotide similarity of contigs within each characterized viral family.

Analysis of the virus-related sequences revealed a diverse range of species spanning different Baltimore classes, including positive-sense single-stranded RNA viruses (ssRNA+), double-stranded RNA viruses (dsRNA), and double-stranded DNA viruses (dsDNA) (Figure 2 and Supplementary Table S2). Among the putative novel viral sequences, eighteen exhibited conserved domains such as polymerase, helicase, and capsid. In contrast, the remaining sequences did not show conserved motifs, likely due to their shorter length or lack of a significant number of related sequences (Supplementary Figure S2).

#### 3.2.1. Characterization of Known Viral Species

Fourteen contigs exhibited high nucleotide sequence identity to the genomes of known viruses and encoded proteins with the same conserved domains as their closest hits. Among these, five contigs ranging from 530 to 1022 nt were related to three distinct strains of the Betaflexivirus *Capillovirus mali*, containing open reading frames (ORFs) for the polymerase, movement protein, and capsid. Notably, two complete ORFs of *Cacao leafroll virus*, a polerovirus, were assembled, encoding the RNA-dependent RNA polymerase (RdRP), Luteovirus P0 protein, and a peptidase. The remaining assembled contigs exhibited similarity to distinct viral sequences. One contig was related to the Red-mite-associated cystovirus, containing ORFs encoding an RdRP (632 nt) and a hydrolase (2353 nt). Additionally, two contigs were related to mitovirus sequences, with one matching the RdRP of *Plasmopara viticola* lesion-associated mitovirus 43 (1783 nt) and the other aligning to the RdRP of *Tongren Botou tick virus 1* (1254 nt) (Supplementary Table S2).

The last contig of 9498 nt showed high identity to the *Potato virus Y* (PVY). It contained a complete ORF of 8214 nt, encoding a polyprotein with conserved domains associated with the polymerase, helicase, coat protein, and peptidase. However, all libraries containing PVY sequences originate from the BioProject PRJEB35419, which includes a total of 1818 high-similarity contigs matching *Solanum tuberosum* and an additional 1159 contigs associated with the Solanum genus. The substantial presence of Solanum-derived contigs suggests a potential contamination in these samples, and Supplementary Figure S3 exhibits more details of this pattern in all libraries that had contigs aligned with *S. tuberosum* sequences. The source of *S. tuberosum* remains unknown, such as the origin of the Potyvirus found, and further investigations are necessary.

#### 3.2.2. Characterization of Novel Viral Species

Overall, 20 contigs showed sequence similarity to known viral species only at the amino acid level. Among these putative viral sequences, 11 contained conserved domains encoding for the polymerase/polyprotein, while the remaining contigs either displayed other domains or lacked recognizable domains and were therefore not subjected to phylogenetic analysis. Hence, six transcripts related to replicative sequences were associated with viruses within the Kitrinoviricota phylum and four with viruses from the Pisuviricota. Additionally, one contig exhibited similarity to dsDNA caulimoviruses, including *Cacao yellow vein banding virus*.

*Kitrinoviricota*

The *Deltaflexiviridae* family is a group of fungi- and plant-infecting viruses characterized by an ssRNA+ polyadenylated genome, typically ranging from 6 to 8.3 kb in size and containing four to five open reading frames. The genome encodes essential proteins such as the RNA-dependent RNA polymerase, viral helicase, and methyltransferase [61,62]. In our study, four putative new deltaflexivirus sequences were assembled, with their genomes ranging in size from 527 to 4432 nt.

The largest contig, measuring 4432 nt, although differing in genome size from previously established *Deltaflexiviridae* members, was assembled with a complete genome structure. It features a complete polyprotein ORF of 2199 nt, which encodes the polymerase and helicase domains, as well as three smaller ORFs that lack conserved domains, which is expected for this family. In contrast, the 3741-nt-long contig only contained the polyprotein ORF (3273 nt), which displayed the polymerase and helicase conserved domains. The two smaller contigs each contained an incomplete polyprotein ORF, ranging from 468 to 513 nt, yet both displayed the polymerase domain as their best hits (Supplementary Figure S2 and Supplementary Table S2).

**Figure 3:**
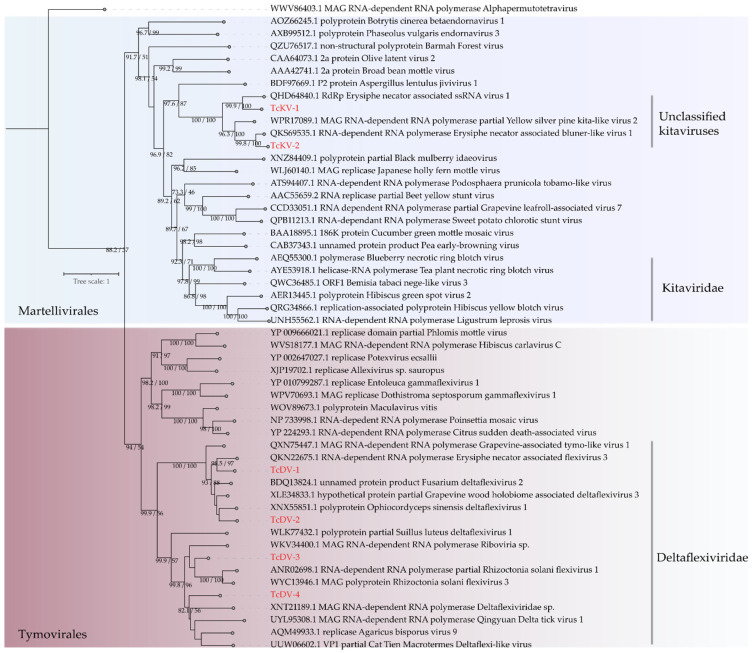
Phylogenetic analysis of Kitrinoviricota viruses. A maximum likelihood phylogenetic tree was constructed based on full-length amino acid sequences of the RNA-dependent RNA polymerase (RdRp). The tree was generated using IQ-TREE, employing the Blosum62+F+R5 evolutionary model with 1000 bootstrap pseudoreplicates for statistical support. The tree was rooted using the RdRp sequence from Alphapermutetravirus (family *Alphapermutetraviridae*) as the outgroup for comparison. The putative new viral species are highlighted in red.

Of note, the phylogenetic analysis clustered the two largest contigs with characterized viruses from the genus Deltaflexivirus, while the two smaller ones were grouped with unclassified members of the *Deltaflexiviridae* family, potentially due to their shorter genome lengths (Figure 3). The sequences were named Theobroma cacao-associated deltaflexivirus 1-4 (TcDV-1-4).

The *Kitaviridae* family comprises plant-infecting viruses with ssRNA+ genomes. Their genomes are multipartite, consisting of two to four segments depending on the viral genus, with at least one large open reading frame typically ranging from 5 to 8 kb. In addition to the replication-associated protein, their genomes encode movement proteins and structural proteins [63].

Two assembled transcripts exhibited amino-acid-level similarity to *Kitaviridae* members. The first, measuring 623 nt, contained an incomplete ORF of 588 nt, while the second, at 3666 nt, harbored a complete ORF of 3378 nt. Notably, both included conserved domains associated with the polymerase, whereas their top BLAST hits lacked identifiable conserved domains (Supplementary Figure S2 and Supplementary Table S2). Phylogenetic analysis using the RdRp ORFs, consistent with the similarity analysis, indicated that the assembled contigs belong to the *Kitaviridae* family, as they clustered with unclassified members of this family. Consequently, these sequences were named Theobroma cacao-associated kitavirus 1 and 2 (TcKV 1-2) (Figure 3).

*Pisuviricota*

**Figure 4:**
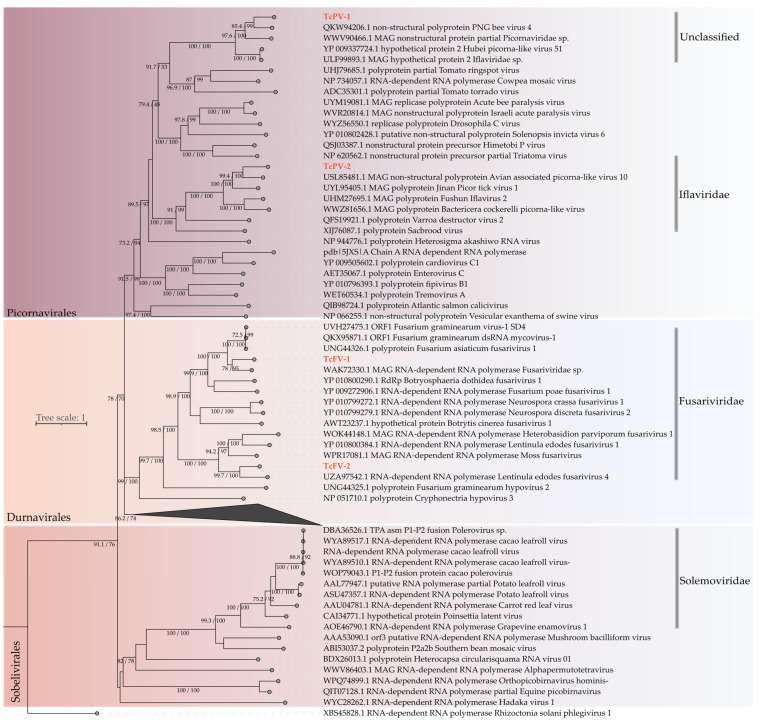
Phylogenetic analysis of Pisuviricota viruses. A maximum likelihood phylogenetic tree was constructed based on full-length amino acid sequences of the RNA-dependent RNA polymerase (RdRp). The tree was generated using IQ-TREE, employing the Blosum62+F+R5 evolutionary model with 1000 bootstrap pseudoreplicates for statistical support. The tree was rooted using the RdRp sequence from Rhizoctonia solani phlegivirus 1 (family *Phlegiviridae*) as the outgroup for comparison. The putative new viral species are highlighted in red.

The *Fusariviridae* family consists of positive-sense, single-stranded RNA viruses with genome sizes varying between 5.9 and 10.7 kb. Their genomes are typically bicistronic, with the larger ORF encoding an RNA-dependent RNA polymerase (RdRp) and an RNA helicase, while the function of the second ORF remains unclear. These viruses are primarily found in fungal hosts, though some evidence suggests that they may also infect oomycetes [64]. In our study, two sequences exhibited similarity to fusaviruses. The first, measuring 6556 nt, contained two complete ORFs of 4758 nt and 1296 nt. The larger ORF encoded the RdRp and a viral helicase, while the second ORF lacked conserved domains, which is characteristic of *Fusariviridae* members (Supplementary Figure S2). The second contig of 1008 nt featured an incomplete ORF of 963 nt that retained the polymerase domain. Based on phylogenetic analysis, these sequences were designated as Theobroma cacao-associated fusarivirus 1-2 (TcFV1-2) (Figure 4).

The order Picornavirales comprises a diverse group of ssRNA+ viruses that infect a wide range of hosts, including plants, invertebrates, and vertebrates. Their genomes typically range from 6 to 12 kb and are characterized by a single large open reading frame encoding a polyprotein with RdRp, helicase, and protease-related domains. This order includes several well-known families, such as *Picornaviridae*, *Secoviridae*, and *Dicistroviridae* [65]. In this study, two assembled transcripts were identified as related to viruses from the Picornavirales order, with lengths of 1331 nt and 1520 nt. Both contigs contained complete polymerase ORFs of 936 nt and 1314 nt, respectively, and notably, they presented a Picornavirales-specific polymerase domain as their best hit (Figure 4).

In the phylogenetic analysis, the larger sequence clustered in a clade of unclassified Picornavirales members, while the second contig grouped with members of the *Iflaviridae* family. Based on these findings, the sequences were designated Theobroma cacao-associated picorna-like virus 1 and 2 (TcPV-1 and TcPV-2).

*Caulimoviridae*

The *Caulimoviridae* family consists of dsDNA viruses that infect plants, often causing persistent infections. Their genomes, typically ranging from 7 to 9.8 kb, are circular and pararetroviral. The genome organization includes multiple open reading frames encoding proteins such as the reverse transcriptase (RT), coat protein, movement protein, and transcriptional regulators. Members of this family are non-enveloped and form icosahedral or bacilliform virions, depending on the genus [66]. A *Caulimoviridae*-related incomplete contig was assembled, measuring 2074 nt. It contained one ORF of 1377 nt, encoding the reverse transcriptase and ribonuclease H domains, respectively.

**Figure 5:**
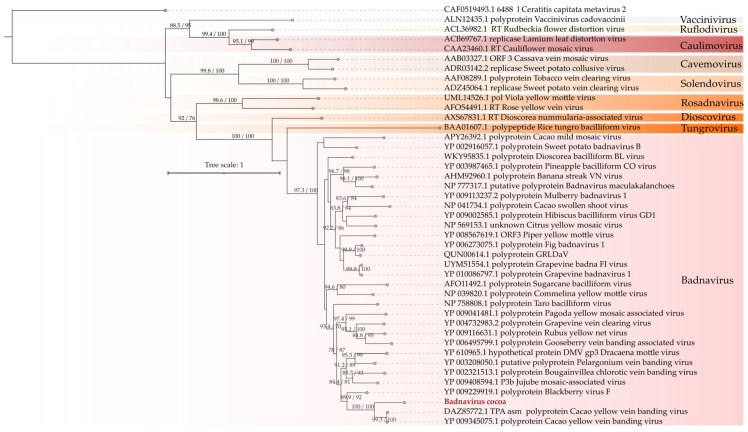
Phylogenetic analysis of the caulimovirus. A maximum likelihood phylogenetic tree was constructed based on full-length amino acid sequences of the reverse transcriptases (RTs). The tree was generated using IQ-TREE, employing the LG+F+R6 evolutionary model with 1000 bootstrap pseudoreplicates for statistical support. The tree was rooted using the RT from *Ceratitis capitata metavirus 2* (family *Metaviridae*) as the outgroup for comparison. The putative new viral species is highlighted in red.

Phylogenetic analysis revealed that this putative viral sequence clustered with members of the Badnavirus genus, notably within the same clade as the well-known cacao pathogen *Cacao yellow vein banding virus* (Figure 5). Interestingly, it was distinct from other known cacao-infecting badnaviruses, such as *Cacao swollen shoot virus* and *Cacao mild mosaic virus*. Under these observations, this sequence was designated as Badnavirus cocoa (BCV).

Of note, nucleotide similarity analysis showed relatively low identity between BCV and the endogenous *Theobroma cacao* viral sequences, specifically Theobroma cacao bacilliform virus 1, with 66.12% identity and 46% coverage.

### 3.3. Wide Spread of Known Pathogens and Novel Theobroma cacao-Associated Viruses

The *Caulimoviridae* family comprises dsDNA viruses that infect plants, often establishing persistent infections. In this context, we investigated the abundance of our viral sequences compared to known Badnavirus species. Endogenous viral elements (EVEs) from this genus have already been reported in the *Theobroma cacao* genome [67]. Notably, in our data, a sequence from *Cacao swollen shoot virus* (CSSV) was detected in 47 different libraries from distinct BioProjects through read quantification analysis (Supplementary Figure S4). However, the alignment of assembled contigs did not reveal any sequence with similarity to this viral species, likely due to its similarity to known endogenous viruses.

Other known viral sequences were also mapped to libraries where no sequences had previously been identified through contig assembly, including *Cacao leafroll virus*. A total of fifteen different libraries from distinct BioProjects were mapped to the complete genome of this virus; only four of them contained metatranscriptomic contigs aligning with the viral sequences.

**Figure 6:**
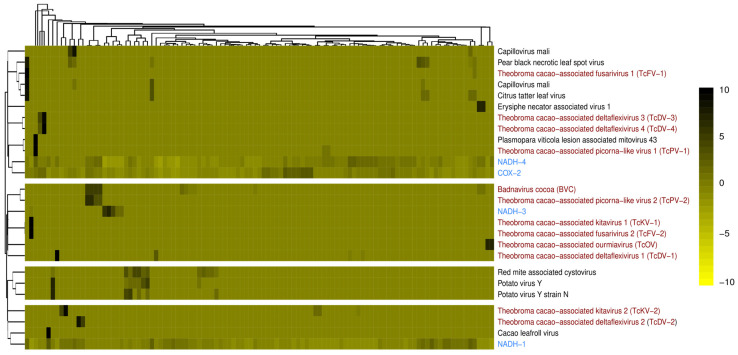
Transcriptional activity of *Theobroma cacao* RNA-seq libraries, highlighting both known and newly identified viral sequences. Novel viral sequences detected in *T. cacao* libraries are indicated in red. Expression profiles of reference cocoa genes across all 109 analyzed libraries are shown in blue. Known and new viral species are represented in black and red, respectively.

The abundance of constitutive genes was higher than that of any known or novel viral sequence characterized in this study. Given the large number of processed libraries and their intrinsic specificities and the different characteristics of each viral sequence within the dataset, only a few clusters allow for the analysis of sequence quantification variability across samples (Figure 6). In general, the detection of the novel viral species was restricted to libraries associated with the same BioProject and geographic region of the library where the virus was first found. In contrast, the distribution of viral families exhibited a broader geographic distribution. Novel species of the *Fusariviridae* family were predominantly found in the Asian continent, particularly in India and China. *Deltaflexiviridae* species were detected in both the USA and Ecuador, while novel *Kitaviridae* sequences were identified in samples derived from the USA and India. However, it is important to point out that we were not able to determine a core virome for *Theobroma cacao*.

## 4. Discussion

The metatranscriptomic analysis of all libraries reveals common patterns characteristic of the relatively unexplored *Theobroma cacao* leaf microbiome [68,69]. Most studies on this subject have primarily focused on the cocoa bean microbiome during the fermentation process [70,71,72,73,74,75], and a few reported the diverse associated virome related to the cocoa microbiome [76,77]. Viral diversity and surveillance in *Theobroma cacao* remain relatively unexplored, as most studies have primarily focused on members of the Badnavirus and Polerovirus genera. This study aimed to comprehensively explore the total virome and its diversity using a public dataset of 109 RNA-seq libraries collected under different conditions, across various regions, and from multiple research projects. We were able to identify multiple viral sequences, and although in some cases they do not represent complete viral genomes, they present many characteristics of viral sequences, such as large open reading frames and the presence of conserved domains associated with viral species. However, these putative viral sequences might originate from symbiotic/pathogenic fungi or microbial communities in different plant tissues, requiring further experiments to determine the possible host.

Despite the unequal number of greenhouse-derived samples compared with wild accessions (only 7.33% of wild accessions libraries), there was no significant variation in the metatranscriptomic profile between these groups. However, due to the large difference in sample size, there is still a bias in the comparative analysis. Notably, only 11% of the samples originate from cocoa pod sequencing, specifically from BioProjects PRJNA613342 (China) and PRJNA604260 (India), derived from lab samples. These libraries exhibited novel cocoa-associated viruses and a high prevalence of fungal contigs. The first BioProject, which analyzed samples from China [78], appears to provide the first report of *Capillovirus mali* sequences in a *T. cacao* RNA-seq library. Commonly known as *Apple stem grooving virus* (ASGV) and *Citrus tatter leaf virus* (CTLV), this virus has been reported to have a global distribution. As for ASGV identification, *Capillovirus mali* has been documented infecting a broad range of hosts, including apples, pears, citrus, kiwifruit, and various other horticultural crops [79,80,81]. Meanwhile, CLTV has been widely recognized as a significant pathogen affecting citrus production worldwide [82]. Currently, there is no evidence based on the literature of *Capillovirus mali* infecting cocoa trees. Similarly, the other known viruses found in our analysis could be associated with a diverse host range inside the cocoa’s microbial community, from symbiotic/pathogenic fungi or aphids and other insects that could have landed on cacao plants after interacting with virus-infected hosts. However, knowledge of these viruses is important to monitor possible spillover events that could occur from these interactions.

A newly characterized putative virus-like sequence from the *Fusariviridae* family, Theobroma cacao-associated fusarivirus 1 (TcFV-1), was identified in the same BioProject, specifically in the RNA-seq library under the SRA code SRR11389078. Although TcFV-1 is a fungal virus, the microbiome analysis of this library reveals a low abundance of fungal reads, with only 0.56% of the total metatranscriptome aligning with fungal targets. This contrasts with the host sample of TcFV-2, identified in SRR11060111, a library from India, which exhibits approximately 11.8% of total metatranscriptomic reads associated with fungal elements. The key commonality between the samples hosting TcFV-1 and TcFV-2 is the plant tissue used for library preparation—the cocoa pod.

The two infected libraries also share the same most abundant fungal genus, with *Fusarium* being the dominant fungal taxon in read alignment and quantification, followed by *Colletotrichum*. Members of *Fusarium* are well-known plant pathogens and have been previously reported to harbor a diverse range of dsRNA mycoviruses [83], particularly *Fusariviridae* infections in *Fusarium graminearum* by *Fusarium graminearum virus 1 strain DK21* (FgV1-DK21) [84,85]. Notably, read alignment and quantification in both libraries hosting TcFV-1 and TcFV-2 indicate the presence of *F. graminearum*, further supporting its association with these viral sequences.

The comprehensive microbiome analysis across all samples revealed a vast and diverse community of fungal and oomycete species within the endophytic environment of *Theobroma cacao*. One likely factor contributing to this high microbial diversity is the cultivation of cocoa in tropical and humid agricultural regions, which provide optimal conditions for the proliferation of fungal phytopathogens [86,87]. Despite the successful spread of these fungal pathogens in *T. cacao*, their uncontrolled proliferation does not go unnoticed. Mycoviruses, or fungal viruses, are widely distributed across diverse fungal taxa, including yeasts, mushrooms, and pathogenic fungi that infect plants, insects, and humans. These viruses are classified into multiple families based on their genomic composition, notably, the majority of mycoviruses possess RNA genomes, with those carrying +ssRNA being assigned to 11 distinct families, including *Alphaflexiviridae*, *Barnaviridae*, *Botourmiaviridae*, *Deltaflexiviridae*, *Endornaviridae*, *Gammaflexiviridae*, *Hadakaviridae*, *Hypoviridae*, *Mitoviridae*, *Narnaviridae*, and *Yadokariviridae* [88]. Within the family *Deltaflexiviridae*, the sole genus, Deltaflexivirus, includes viruses with both segmented and unsegmented genomes [89]. A conserved feature among all Deltaflexivirus members is the presence of a gene encoding an RNA replicase, which plays a crucial role in viral replication and maintenance [90].

Our analysis successfully identified four novel *Deltaflexiviridae* members in *Theobroma cacao* samples from Ecuador and the USA projects. This viral family was recently proposed by Kunfei et al. [91], who investigated *Sclerotinia sclerotiorum*, an important plant pathogenic fungus known to harbor a diverse range of mycoviruses. The newly identified viral sequences, Theobroma cacao-associated deltaflexivirus 1–4 (TcDV-1 to TcDV-4), were detected in three distinct BioProjects: PRJNA558793, PRJNA326055, and PRJNA785999. While the first two projects focused on the comparative analysis of genetic variants and domesticated trees, BioProject PRJNA785999 aimed to evaluate resistant and susceptible cocoa genotypes following *Phytophthora palmivora* inoculation. Notably, there are no known reports of *Deltaflexiviridae* members infecting *P. palmivora*. However, other Phytophthora species have been documented as hosts for viruses from the *Narnaviridae*, *Totiviridae*, *Endornaviridae*, *Megabirnaviridae*, and *Tombusviridae* families, as well as members of the order Bunyavirales [92,93,94].

The viral contigs TcDV-3 and TcDV-4 were identified in the SRA library SRR17587555, a *Phytophthora palmivora*-inoculated sample from the CCN51 genotype. Metatranscriptomic analysis of the assembled contigs in this library revealed that 56.9% of the contigs aligned with high-similarity protein sequences from the *Phytophthora* genus, while approximately 9.2% aligned with high-similarity targets from the *Moniliophthora* genus. The substantial presence of *Phytophthora* contigs, combined with the documented submitted inoculation conditions, suggests a potential pathogen–host interaction between TcDV-3/TcDV-4 and *P. palmivora*.

The sample infected with TcDV-1 exhibited 13% assembled contigs with high identity to sequences derived from species within the Moniliophthora genus in the total metatranscriptomic analysis. In contrast, TcDV-2 did not show significant fungal diversity, with only two contigs aligning to *Sphaceloma batatas*, a known phytopathogenic fungus. The sample containing TcDV-2 belongs to BioProject PRJNA558793, where another novel viral sequence, Theobroma cacao-associated kitavirus 2 (TcKV-2), was also identified.

Members of the *Kitaviridae* family are known to infect a wide range of host plants globally, causing economically significant diseases in crops such as citrus, tomato, and blueberry. These viruses are transmitted by mites of the Brevipalpus genus [63,95]. Recently, Brevipalpus mites have been reported infecting *Theobroma cacao* in Peru [96], and historical data indicate their global widespread distribution [97,98]. The libraries infected with TcKV-1 and TcKV-2 originate from India and the USA, respectively. Despite the geographical distance between these samples, it is plausible to speculate that Brevipalpus mites could infect *T. cacao* in other regions and potentially serve as vectors for these novel kitaviruses.

The recently identified *Cacao leafroll virus* (CaLRV), a member of the Polerovirus genus within the *Solemoviridae* family, was also detected in our analysis. CaLRV was first described by Adegbola et al. (2023) [99] as “cacao polerovirus” and was subsequently fully characterized by the same research group in 2024 [67]. The samples containing CaLRV originate from BioProject PRJNA558793, which focuses on investigating disease resistance in *Theobroma cacao* through whole-genome and transcriptome sequencing of diverse genotypes.

The Badnavirus cocoa (BVC) was identified as an assembled contig in samples SRR9203102, SRR9203103, and SRR3217279 from French Guiana. These samples belong to BioProjects PRJNA326055 and PRJNA314774, both of which aimed to perform comparative analyses on the effects of domestication on genome evolution across different crop species and wild genotypes. Quantification analysis detected this viral sequence in a total of 11 RNA-seq libraries, encompassing additional geographic regions such as the USA and Colombia, but at lower TPM expression levels compared to the contigs from French Guiana.

Badnaviruses are known to integrate multiple sequences into the *Theobroma cacao* genome, with these integrations varying in type and prevalence among different genetic groups. This suggests that viral integration occurred before or during species diversification [100]. Recent studies have surveyed diverse cacao germplasm, identifying multiple insertions of varying lengths and orientations. Notably, these endogenous viral elements (EVEs) can interfere with host gene expression, potentially impacting plant performance [33]. The high diversity of integrated sequences, coupled with the limited characterization of different genotypes, highlights the largely unexplored nature of EVEs in the *T. cacao* genome. This may explain the observed quantification of BVC sequences in certain samples without direct assembly or alignment to known viral or EVE reference sequences.

While the exploration of public data is crucial, this approach is inherently accompanied by several limitations, such as incomplete metadata, potential biases in the sample origins (e.g., geographical location or tissue type), and the inability to perform experimental validation. Despite encountering some of these challenges in our study, we sought to present robust evidence through comprehensive bioinformatics analyses, which we believe substantiate the presence of viral sequences linked to cocoa-derived samples available in the NCBI SRA public database.

## 5. Conclusions

This study advances our understanding of the *Theobroma cacao* virome by uncovering a diverse array of previously uncharacterized viral sequences. The identification of eleven novel viruses, including a new Badnavirus, highlights the hidden complexity of cocoa-associated viruses, providing new insights into their diversity and ecological roles and interactions within the plant microbiome. The detection of *Capillovirus mali* in cocoa suggests a potential new host for this species, emphasizing the need for further surveillance and prospecting for emerging viral threats. Additionally, the discovery of novel mycoviruses reveals the intricate dynamics of cocoa-associated microbial communities, underscoring the complexity of plant–virus–microbiome interactions. These findings reinforce the importance of virome studies in agricultural systems, particularly for economically significant crops like cocoa, where viral infections pose a major threat to productivity. By expanding the catalog of cocoa-associated viruses, this research contributes to enhanced disease monitoring and management strategies, ultimately supporting sustainable cocoa production and the resilience of global supply chains.

## Data Availability

The data presented in this study are contained within the article and Supplementary Materials. Additional data may be available from the corresponding author upon reasonable request.

## Supplementary Materials

The following supporting information can be downloaded at: https://www.mdpi.com/article/10.3390/v17050624/s1, Figure S1: t-SNE based on the relative abundance of viral species; Figure S2: Structural annotation of the novel viruses; Figure S3: Frequence of *S. tuberosum* contigs in *T. cacao*-derived samples; Figure S4: Quantification of previously known cocoa viruses; Table S1: Samples metadata; Table S2: Viral sequences characterization; Table S3: Overview of transcripts quantification.
