# Supplementary material for: Theobroma cacao Virome: Exploring Public RNA-Seq Data for Viral Discovery and Surveillance
Source: Viruses. 2025 Apr 26;17(5):624. doi: 10.3390/v17050624 (PMC12115555; doi:10.3390/v17050624)
Supplement: Supplementary file 1 [file viruses-17-00624-s001.zip › viruses-3560589-supplementary/Supplementary-Figure S2.pdf]

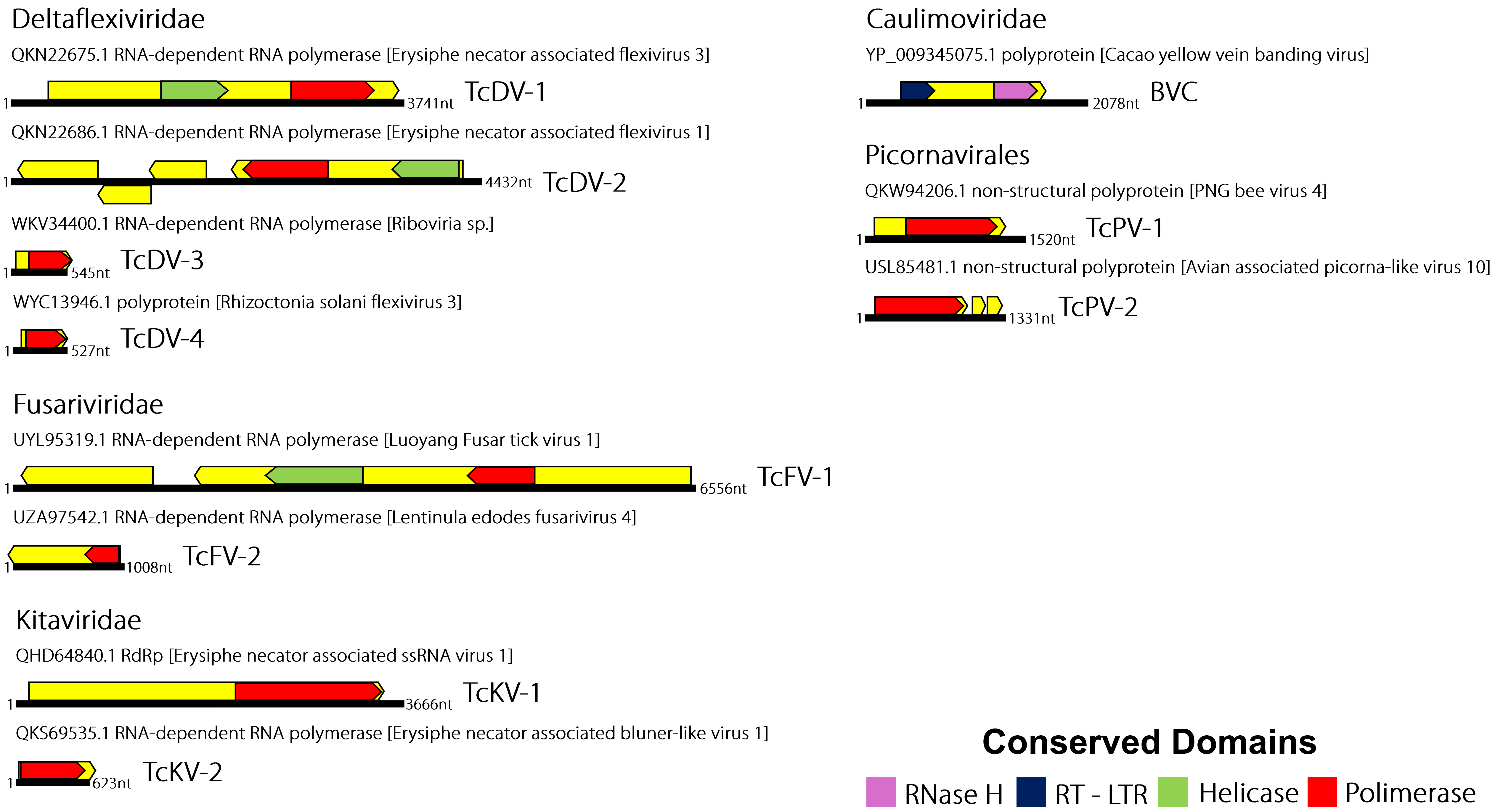
Deltaflexiviridae
QKN22675.1 RNA-dependent RNA polymerase [Erysiphe necator associated flexivirus 3]
1
3741nt
TcDV-1
QKN22686.1 RNA-dependent RNA polymerase [Erysiphe necator associated flexivirus 1]
1
4432nt
TcDV-2
WKV34400.1 RNA-dependent RNA polymerase [Riboviria sp.]
1
545nt
TcDV-3
WYC13946.1 polyprotein [Rhizoctonia solani flexivirus 3]
1
527nt
TcDV-4
Fusariviridae
UYL95319.1 RNA-dependent RNA polymerase [Luoyang Fusar tick virus 1]
1
6556nt
TcFV-1
UZA97542.1 RNA-dependent RNA polymerase [Lentinula edodes fusarivirus 4]
1
1008nt
TcFV-2
Kitaviridae
QHD64840.1 RdRp [Erysiphe necator associated ssRNA virus 1]
1
3666nt
TcKV-1
QKS69535.1 RNA-dependent RNA polymerase [Erysiphe necator associated bluner-like virus 1]
1
623nt
TcKV-2
Caulimoviridae
YP_009345075.1 polyprotein [Cacao yellow vein banding virus]
1
2078nt
BVC
Picornavirales
QKW94206.1 non-structural polyprotein [PNG bee virus 4]
1
1520nt
TcPV-1
USL85481.1 non-structural polyprotein [Avian associated picorna-like virus 10]
1
1331nt
TcPV-2
Conserved Domains
RNase H
RT - LTR
Helicase
Polimerase
