# Supplementary figures and images for: Theobroma cacao Virome: Exploring Public RNA-Seq Data for Viral Discovery and Surveillance
Source: Viruses. 2025 Apr 26;17(5):624. doi: 10.3390/v17050624 (PMC12115555; doi:10.3390/v17050624)

# Species relative abundance

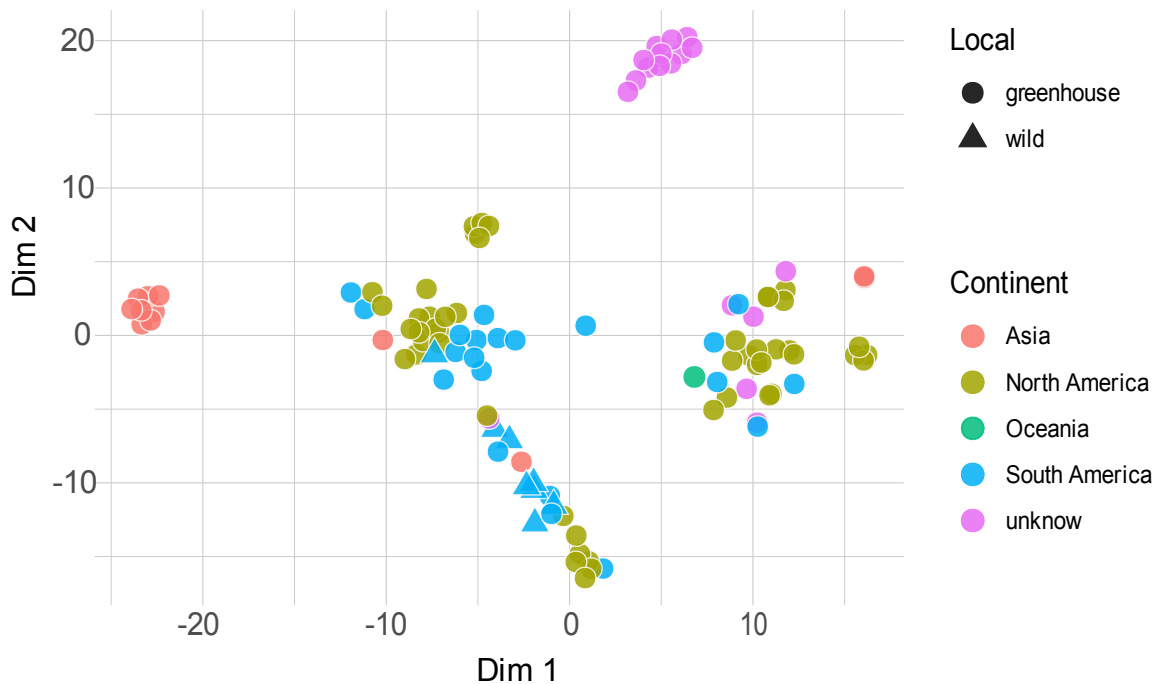

Supplement: Supplementary file 1 [file viruses-17-00624-s001.zip › viruses-3560589-supplementary/Supplementary-Figure S1.pdf]

**Frequency of *S. tuberosum* contigs in samples**

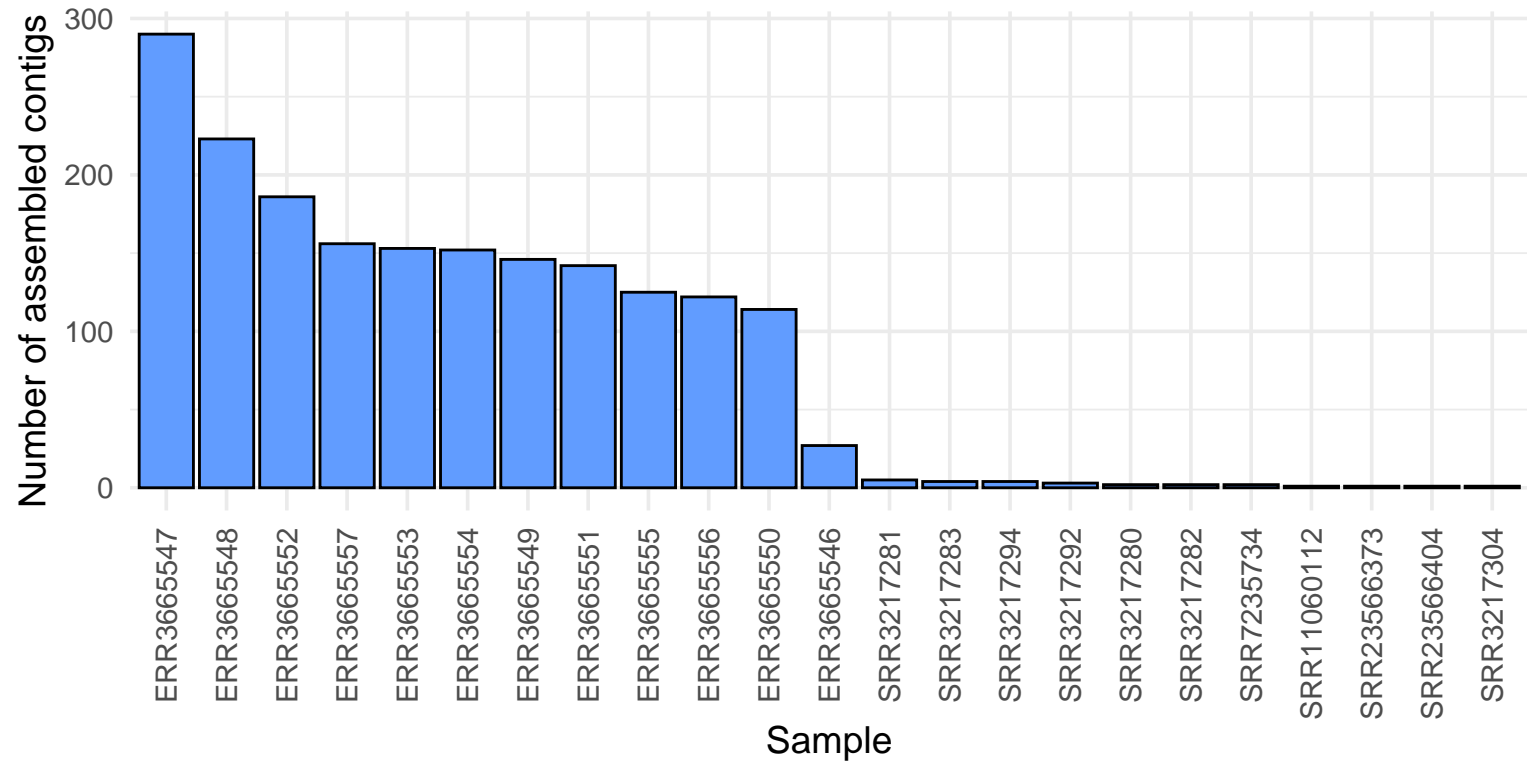

Supplement: Supplementary file 1 [file viruses-17-00624-s001.zip › viruses-3560589-supplementary/Supplementary-Figure S3.pdf]

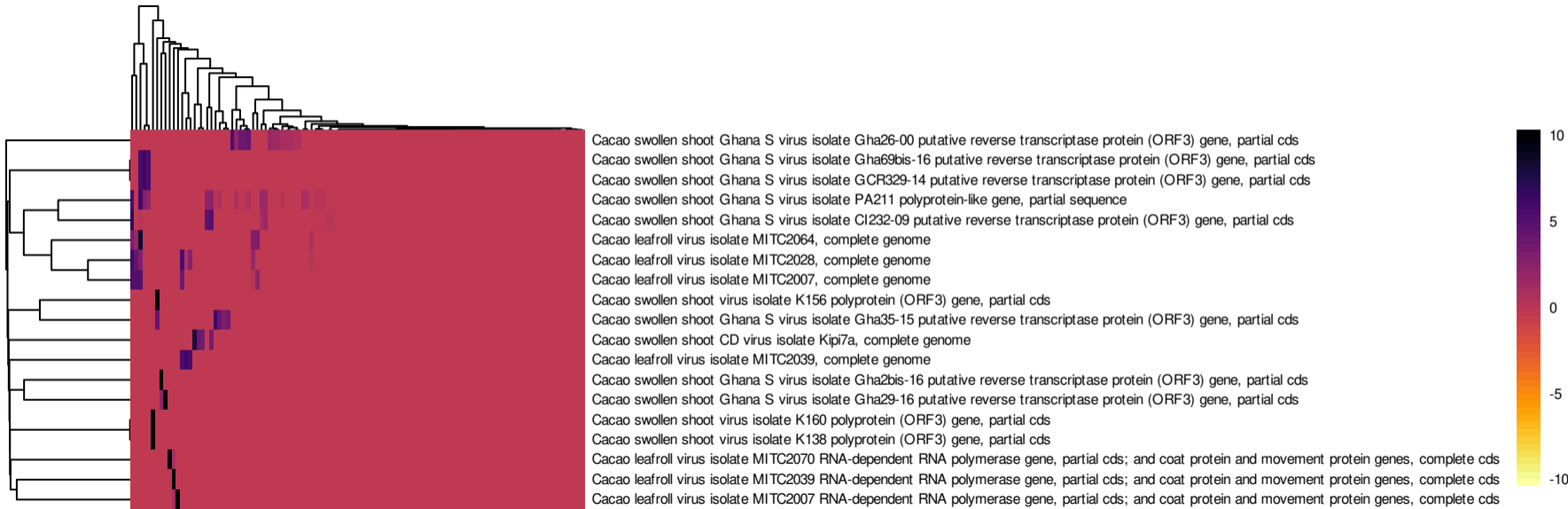

Supplement: Supplementary file 1 [file viruses-17-00624-s001.zip › viruses-3560589-supplementary/Supplementary-Figure S4.pdf]
